# Clinical and molecular characterization of familial hypocalciuric hypercalcemia in an endocrine practice: a case series of 25 patients

**DOI:** 10.1093/jbmrpl/ziag049

**Published:** 2026-03-26

**Authors:** Jack Lin, Tracy S Wang, Amy Donahue, Joseph L Shaker

**Affiliations:** Department of Medicine, Endocrinology and Molecular Medicine, Medical College of Wisconsin, Milwaukee, WI 53226, United States; Department of Surgery, Medical College of Wisconsin, Milwaukee, WI 53226, United States; Department of Human Genetics, Medical College of Wisconsin, Milwaukee, WI 53226, United States; IntrospectDNA, LLC, Milwaukee, WI 53212, United States; Department of Medicine, Endocrinology and Molecular Medicine, Medical College of Wisconsin, Milwaukee, WI 53226, United States

## Abstract

Familial hypocalciuric hypercalcemia (FHH) is a disorder caused by abnormal sensing of calcium by the parathyroids and renal tubules and may mimic primary hyperparathyroidism (PHPT). Patients with FHH should not be subjected to unnecessary and potentially harmful parathyroid surgery. We sought to evaluate the clinical and biochemical presentation of FHH in our endocrine practice. This was a retrospective chart review done after Institutional Review Board approval. We describe 25 cases of likely FHH from our endocrine practice (20 FHH1, 1 FHH2, and 4 FHH3). The 24-h urinary calcium levels were often greater than 100 mg/24 h and sometimes greater than 200 mg/24 h. The calcium to creatinine clearance ratio (CCCR) was consistently <0.01 in 10 of 22 (45%). We also describe several variants of *CASR* and 1 variant of *GNA11* that are probably pathogenic. Some patients, particularly FHH3 patients, have hypercalcemia severe enough to warrant treatment with a calcimimetic medication (cinacalcet). Traditional methods of excluding FHH using 24-h urinary calcium and CCCR are not adequate to distinguish FHH and PHPT. In addition to young age, family history of hypercalcemia and low CCCR, we believe molecular testing should be considered prior to surgery when hypercalcemia cannot be demonstrated to be acquired and in the case of persistent PHPT after surgery by an experienced parathyroid surgeon.

## Graphical Abstract

**Graphical Abstract:**
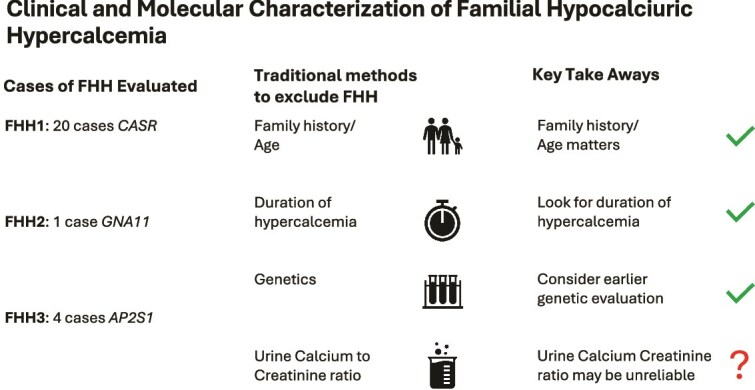

## Introduction

Primary hyperparathyroidism (PHPT) is a relatively common endocrinopathy usually caused by solitary adenomas (~85% to 90%),^1^ less frequently multiple gland disease (~10% to 15%)^1^ and rarely by parathyroid cancer.^2^ Inherited forms of PHPT may account for up to 15% of cases.^1^ These include syndromic familial conditions, such as multiple endocrine neoplasia (MEN) 1, caused by pathogenic loss-of-function variants of the *MEN1* tumor suppressor gene; MEN2A and 2B, both caused by pathogenic loss-of-function variants of the *RET* proto oncogene; MEN4, caused by pathogenic loss-of-function variants of the *CDKN1B* tumor suppressor gene; and the hyperparathyroidism-jaw tumor syndrome, caused by pathogenic loss-of-function variants of the *CDC73* tumor suppressor gene. Additionally, there are non-syndromic forms of inherited PHPT (familial isolated PHPT), with pathogenic loss-of-function variants in *MEN1*, *CDC73*, and *CASR* responsible for about 30% of the affected families,^1^ as well as familial hypocalciuric hypercalcemia (FHH), described below. Recently, gain-of-function variants in *GCM2* have been found to predispose to PHPT (loss-of-function variants are known to cause dominant and recessive hypoparathyroidism).^1^ Among the familial conditions, it is very important to distinguish PHPT from FHH, a disorder caused by abnormal sensing of calcium by the parathyroids and renal tubules, as patients with FHH should generally not undergo parathyroid surgery.^1^

Familial hypocalciuric hypercalcemia (also known as familial benign hypercalcemia) was first described by Jackson and Boonstra in 1966^3^ and more clearly defined by Marx et al.^4^^,^^5^ and Attie et al.^6^ Classically, these patients have lifelong hypercalcemia with normal PTH levels, along with relative hypocalciuria.^7^ About 24% have elevated PTH levels.^7^ The calcium-sensing receptor gene (*CASR*) was the first gene found to be associated with FHH^8^^,^^9^ and *CASR* pathogenic loss-of-function variants appear to account for approximately 65% of families (FHH1).^1^ Additionally, severe neonatal hyperparathyroidism has been seen in families with FHH^10^ and has been found to be caused by biallelic and sometimes monoallelic pathogenic variants of *CASR.*^11^^,^^12^

**Figure 1:**
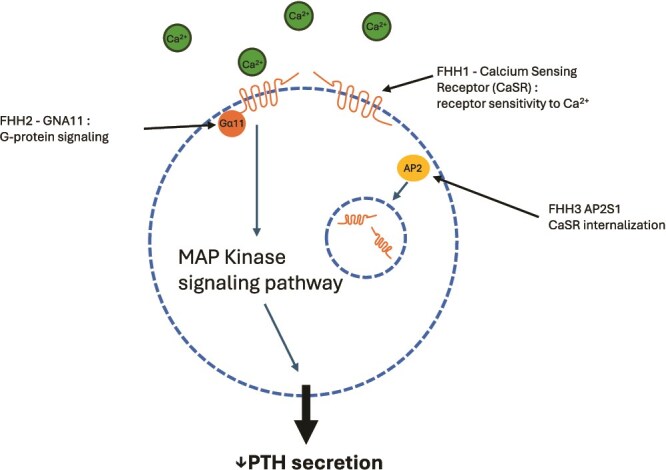
Pathogenic variants in *CASR* results in decreased sensitivity to external calcium (resulting in a higher threshold required to decrease PTH secretion). *GNA11* pathogenic variants result in decreased MAP kinase signaling cascades to the same stimulus. Pathogenic variants in *AP2S1* result in decreased internalization of the CASR.

Loss-of-function variants of *CASR* in parathyroids results in a higher ionized calcium required to normalize PTH.^13^ Further, the loss-of-function of the calcium sensing receptor in the distal renal tubule results in enhanced renal calcium reabsorption.^13^ Subsequently, loss-of-function variants of 2 genes downstream in calcium sensing have been implicated in FHH. Loss-of-function variants of the G-protein subunit α_11_ (*GNA11*)^14^ and adaptor-related protein complex 2, sigma 1 subunit (*AP2S1*)^15^ have been shown to cause FHH2 and FHH3, respectively (Figure 1). *GNA11* encodes a protein which is a signaling partner for the CASR, and *AP2S1* encodes a protein involved in internalization of the CASR.^16^ Pathogenic variants in these 2 genes appear to explain a small additional percentage of families with FHH. There are also documented isolated FHH cases that have been found to be due to de novo mutations^17^ and up to 20% of families with suspected FHH have yet unknown molecular causes.^18^

Classically, the diagnosis of FHH is aided by measurement of the 24-h urinary calcium and creatinine with subsequent calculation of the calcium to creatinine clearance ratio (CCCR) (24-h urinary calcium/serum calcium)/(24-h urinary creatinine/serum creatinine). Life-long hypercalcemia and family history of hypercalcemia are clues to the diagnosis of FHH. Traditional teaching is that a CCCR < 0.01 is consistent with FHH and CCCR > 0.02 is more consistent with PHPT and between these 2 values is indeterminate.^13^ Unfortunately, the specificity and sensitivity of the CCCR leave much to be desired. Christensen et al. studied 54 patients with genetically-proven FHH1 and 97 patients with surgically-cured PHPT.^7^ Using a CCCR of <0.02 picked up 53/54 FHH1 and using <0.01 missed 20% FHH1. The problem is that 35% (34/97) PHPT had CCR <0.02.

Additional reports suggest a CCCR <0.01 is only seen in 70%-80% of patients with FHH and up to 10% of patients with FHH actually have a ratio >0.02.^2^ Beyond that, up to 40% of patients have a ratio between 0.01 and 0.02.^7^ Further, a study of 1000 patients with PHPT suggested that two-thirds of patients with surgically proven PHPT had CCCR <0.02.^19^ Genetic testing of patients for the known genes associated with FHH can serve as an additional tool for evaluation but also has limitations. Variants of unknown significance (VUS) may complicate interpretation of genetic testing, and a negative genetic test result does not rule out FHH as the molecular etiology is unknown (whether due to additional unidentified genes or variants in the known genes that are not included in testing) in up to 20% of cases. Given the typically benign nature of FHH, appropriate identification of as many of these patients as possible using all available information is crucial as failure can lead to inappropriate surgery. Herein, we describe our experience with 25 patients with likely FHH from an academic endocrinology practice experienced in calcium disorders.

## Materials and Methods

This is a retrospective chart review approved by the Medical College of Wisconsin (MCW) Institutional Review Board. Patients were identified from clinician memory as well as a secure list within the EPIC electronic medical system by one of the authors (J.L.S.) and were evaluated 2011-2025. Serum/plasma and urine chemistries were measured by routine methods in clinical laboratories. The reference intervals for calcium and phosphate were 8.4-10.2 mg/dL and 2.5-4.8 mg/dL, respectively. Intact PTH (normal, 15-72 pg/mL) was measured by electrochemiluminescence immunoassay (Roche Diagnostics). 25OHD (optimal, 30-50 ng/mL) was measured using the DiaSorin Liaison Assay (DiaSorin). At our center, measurement of 24-h urinary calcium and creatinine is routine in the evaluation of apparent PHPT. The CCCR was calculated as follows: (24-h urinary calcium/serum calcium)/(24-h urinary creatinine/serum creatinine). BMD was measured by DXA. The lowest T-score (postmenopausal women and men older than 50 yr) or Z-score (premenopausal women and men younger than 50 yr) of the LS, FN, TH, or radius 33% site was reported in the table. Clinical molecular genetic testing was ordered for each patient based on factors including availability of genes and patient insurance by a clinician (physician or genetic counselor) and performed by CLIA/CAP certified clinical genetic testing companies, and the genetic test reports were provided to the ordering provider and/or genetic counselor.

## Illustrative cases

### Case 18

#### FHH2

The patient is a 61-yr-old female with a history of hypercalcemia dating back to her 20s. There was a history of uric acid kidney stones and diabetes mellitus type 2. The family history was significant for hypercalcemia in her mother. Further, her sister reportedly had hypercalcemia, high-normal PTH, and low urinary calcium. Laboratory data included calcium levels of 9.9-11 mg/dL, ionized calcium levels of 1.34-1.43 mmol/L, phosphate levels of 2.4-2.5 mg/dL, magnesium levels of 1.8 mg/dL, creatinine of 0.6-1.21 mg/dL, and 25OHD of 25-36 ng/mL. The urinary calcium was 208 mg/24 h and the CCCR was 0.01. Molecular testing for *CASR* and *AP2S1* were negative. One VUS in the *GNA11* gene at position c.646T>A (p.W216R) was detected**.** The variant segregates with 1 affected sibling and 2 unaffected siblings. ClinVar suggests this variant is not observed in large populations^20^ and in-silico analysis predicts deleterious effect.^21^ The analysis from Alpha Missense^22^ suggests this variant is likely pathogenic.

### Case 13

#### PHPT and FHH1

A 55-yr-old woman was seen for persistent hypercalcemia after 2 parathyroid operations. At age 54, she presented with serum calcium levels of 11.2-12.2 mg/dL, intact PTH levels of 93-150 pg/mL, phosphate of 2.4 mg/dL, creatinine of 0.95 mg/dL, and 25OHD of 34 ng/mL. The urinary calcium was 334 mg/24 h and CCCR was 0.018. She underwent parathyroid surgery and had a 920 left superior parathyroid adenoma removed (pathology revealed hypercellular parathyroid tissue). Her PTH decreased to 18.5 pg/mL immediately postoperatively, however, persistent hypercalcemia (10.5-11.7 mg/dL) was found within 1 mo of surgery. The PTH was 21-82 pg/mL, urinary calcium was 126 mg/24 h, and CCCR was 0.01. Six-months after the first surgery, she had repeated parathyroid surgery with 2.5 parathyroids removed (no pathologic abnormality). Mild hypercalcemia persisted with calcium 10.3-10.6 mg/dL and ionized calcium was 1.32 and 1.37 mmol/L. The phosphate was 2.5 and 2.9 mg/dL, magnesium was 2.2 mg/dL, creatinine was 0.8-0.93 mg/dL, 25OHD was 37 ng/mL, and PTH was 27-47 pg/mL. The urinary calcium was 159 mg/24 h and CCCR was 0.011. There was no history of kidney stones. The family history revealed “borderline hypercalcemia” in her mother and her brother (brother has had kidney transplant). She has a sister with a history of a pituitary tumor.

Molecular analysis of *AP2S1*, *CASR*, *CDC73*, *CDKN1B*, *GNA11*, *MEN1*, and *RET* was performed. A VUS in the *CASR* gene at c.206G>A (p.Arg69His) was found and was later upgraded to likely pathogenic.

### Case 10

#### FHH1 VUS

A 21-yr-old female was evaluated for hypercalcemia dating back to at least age 16. There was a history of cognitive delay and Crohn’s disease. The family history was significant for hypercalcemia in her mother (11.5 mg/dL). Her sister reportedly has hypercalcemia with a low urinary calcium. A maternal aunt and maternal cousin also have hypercalcemia. Laboratory data included calcium 10.6-12.1 mg/dL, ionized calcium 1.56 mmol/L, phosphate 2.4-3.6, magnesium 2.3 and 2.4 mg/dL, 25OHD 16-26 ng/mL, and intact PTH 27-58 pg/mL. The urinary calcium was 205 mg/24 h (no creatinine was done on the urine to calculate CCCR). Molecular testing revealed normal *CDC73* and *MEN 1* genes. A VUS was identified in *CASR* gene (c.566A>G/p.Asn189Ser). This VUS has been reported in at least 1 patient with suspected FHH.^20^ Analysis from Alpha Missense^22^ suggests this variant is likely benign. Genetic testing for FHH2 and FHH3 was not done and the patient was lost to follow-up.

### Case 12

#### FHH3 managed with cinacalcet

A 56-yr-old woman was evaluated for hypercalcemia dating back to approximately age 13. There was a history of DM2 and XXX syndrome. The family history was significant for hypercalcemia in her son. Laboratory data revealed calcium levels of 11.7-13.5 mg/dL, ionized calcium of 1.67-1.71 mmol/L, phosphate of 2.0-3.3 mg/dL, magnesium of 2.3 mg/dL, creatinine of 0.86-1.4 mg/dL, 25OHD of 28-64 ng/mL, and PTH of 129-158 pg/mL. The urinary calcium was 22 mg/24 and CCCR was 0.002. Genetic testing revealed no variants in *CASR*, *CDC73* (*HRPT2*), *CDKN1B*, *MEN1*, *GNA11*, and *RET*. She was heterozygous for a pathogenic variant in the *AP2S1* gene (c.44G>T (p.Arg15Leu)). She was treated with cinacalcet 30 mg twice daily with improvement in serum calcium (10.9-12.1 mg/dL).

### Case 5

#### FHH3 discovered in pregnancy with history of prior parathyroid surgery

A 45-yr-old women was seen for hypercalcemia. She was G1P0 and 26 wk pregnant. Eight-years earlier, she was found to have hypercalcemia with a calcium of 11.2 mg/dL (albumin 4.0 g/dL). Evaluation included PTH levels of 52, 56, and 39 pg/mL. Calcium levels ranged from 10.4 to 12.1 mg/dL, and the ionized calcium levels were 1.45 and 1.57 mmol/L. The phosphate levels were 2.9 and 3.5 mg/dL, magnesium was 2.3 mg/dL, and renal function normal. The urinary calcium was 239 mg/24 h and CCCR was 0.012. Genetic testing at that time revealed no variants in the *MEN1*, *CDC73*, and *CASR* genes. Four-years before presentation to us, she underwent parathyroid surgery (left inferior parathyroid, excision with parathyroid gland and adipose tissue weighing 170 mg). The intraoperative PTH decreased from 56 to 21 pg/mL but hypercalcemia persisted. After surgery, the calcium was 11.3 mg/dL, ionized calcium was 1.49 mmol/L, PTH were 27 and 34 pg/mL, The 25OHD was 21 ng/mL, urinary calcium was 226 mg/24 h, and CCCR was 0.008. Her family history was initially negative for hypercalcemia or parathyroid disorders; however, her sister was later found to have hypercalcemia. Genetic testing was done on *AP2S1*, *CASR*, *CDC73*, *CDKN1B*, *GNA11*, *MEN1*, and *RET*. There was a pathogenic variant in the *AP2S1* gene at (c.43C>T p.Arg15Cys) consistent with FHH3. She was managed with hydration and calcium levels were stable during pregnancy. She delivered a healthy female child (vaginal delivery) at 38 wk and 5 d (Weight: 8 lb 11.5 oz 3955 g). We advised close monitoring of the infants’ calcium level as the infant would be at risk for hypocalcemia if she did not inherit FHH.

**Table 1:** Summary of cases.

**Patient #**	**1**	**2**	**3**	**4**	**5**
**Age/gender**	58M	68F	36F	48F	36F
**Age initial hypercalcemia noted by history or records**	57	30s	28	46	28
**FH**	“Multiple Family Members”	Mother, Brother	No definite Mom calcium 9.9-10.2 mg/dL PTH 109 pg/mL	Sister	Sister
**Calcium (8.4-10.2 mg/dL)**	9.7-12.8^a^	10.0-11.0	9.5-10.9	10.4-10.9	10.8-11.1
**Albumin (3.8-5.0 g/dL)**	3.8-4.4	4.3	3.7-4.5		3.5,3.7
**Ionized calcium** **(1.18-1.33 mmol/L)**	1.36-1.54	1.32-1.43	1.33, 1.28	1.37-1.42	1.43.1.47
**Phosphate (2.5-4.8 mg/dL)**	1.8-3.0	2.8	2.2-3.4	3.4	2.5, 3.4
**Magnesium (1.6-2.6 mg/dL)**	1.4-2.2	2.0	2.1-2.5	2.1	2.2
**Creatinine (0.5-1.1 mg/dL)**	0.61-1.13	0.95-1.1	0.77-1.10	0.69-0.94	0.49-0.78
**PTH (14-72 pg/dL)**	27-163	50-86	60-87	30-51	33, 45
**25(OH)** **vitamin D (30-50 ng/dL)**	16-58	65	21-31	44, 37	25
**Urinary calcium (mg/24 h)**	12 (no creatinine), 91, 251, 10	70	122 108	195 164	239, 226
**CCCR**	0.004, 0.022, 0.0005	0.006	0.004, 0.004	0.013, 0.009	0.012, 0.008
**BMD** **Lowest T or Z**	T-score −0.5	T-score −3.4	Z-score −1.5	Z-score −1.2	
**Kidney stones**	No	No	No	No	No
**Genetic testing**	Pathogenic *CASR* c.73C>T (p.Arg25^*^)	Pathogenic *CASR* **c.**547_548del (p.Phe183Profs^*^7)	Pathogenic *CASR* 1606DelG	VUS *CASR* c.1189G>A p.Gly397Arg (G397R) Later changed to pathogenic	Pathogenic *AP2S1* c.43C>T (p.ARG15CYS)
**Alpha missense**					
**Other genes tested**	*GNA11, AP2S1, MEN1, RET, CDC73, CDKN1B, GCM2*	*GNA11, AP2S1, MEN1, RET, CDC73,CDKN1B,* *GCM2*		*CDC73, MEN1*	*CASR, CDC73, CDKN1B,* *GNA11, MEN1, RET*
**Treated with cinacalcet**	Yes	No	No	No	No
**Miscellaneous**		3.5 gland PTx in her 30s	Scan negative US inconclusive	US negative	FHH Discovered during pregnancy. Failed Ptx @ 32 Single gland removed (170 mg) IOPTH 56 to 21 Hypercalcemia Persisted Preop Scan negative US negative CT possible left inferior adenoma
**Patient #**	**6**	**7**	**8**	**9**	**10**
**Age/gender**	75 M	56F	55F	50M	21F
**Age initial hypercalcemia noted** **By history or Records**	20s	48	52	40	16
**FH**	2 brothers and father	2 sisters and father	Sister of patient 7	15 yo daughter calcium 11, albumin 4.7, PTH 21 ? Sister	Mother (calcium 11.5 mg/dL) Sister “Hypercalcemia with low urinary calcium” Maternal cousin
**Calcium (8.4-10.2 mg/dL)**	Unknown	10.8-11.7	9.6-11.0	9.9-11.1	10.6-12.1
**Albumin (3.8-5.0 g/dL)**	Unknown	4.5-4.8	3.8-4.3	4.2-4.5	3.9-4.5
**Ionized calcium** **(1.18-1.33 mmol/L)**	Unknown	1.46-1.51	1.33-1.46	1.37, 1.40	1.56
**Phosphate (2.5-4.8 mg/dL)**	Unknown	2.4-3.2	1.9	2.6	2.4-3.6
**Magnesium (1.6-2.6 mg/dL)**	Unknown		1.8	2.3	2.3, 2.4
**Creatinine (0.5-1.1 mg/dL)**	Unknown	0.58-0.71	0.59-0.80	0.87-1.04	0.65-0.82
**PTH (14-72 pg/dL)**	Unknown	36-100	57	37-74	27-58
**25(OH)** **Vitamin D (30-50 ng/dL)**	Unknown	31,33	42	24,36	16-26
**Urinary calcium (mg/24 h)**	Unknown	140	326	110 112	205
**CCCR**	Unknown	0.008	0.012	0.005, 0.008	No urinary creatinine done
**BMD** **Lowest T or Z**	Unknown	T-score −2.6	T-Score 0.5		Z score −0.9
**Kidney stones**	No	No	No	No	No
**Genetic testing**	Pathogenic *CASR* c.554G>A (p.Arg185Gln)	Pathogenic *CASR* c.2643_2644ins Alu (p.Lys882fs)	Pathogenic *CASR* c.2643_2644ins Alu (p.Lys882fs)	Initial testing *CASR* negative subsequent pathogenic mutation *CASR* Deletion exons 5-7	VUS *CASR*
**(c.566A>G/p.Asn189Ser)**					
**Alpha missense**					Likely benign
**Other genes tested**	*AP2S1, CDC73, CDKN1B, GCM2, GNA11, MEN1, RET,*	*AP2S1, CDC73, CDKN1B, GNA11, MEN1, RET*		*AP2S1, CDC73, CDKN1B, GNA11, MEN1, RET*	*CDC73, MEN1*
**Treated with cinacalcet**	No	No	N0	No	No
**Miscellaneous**	PTx × 4 late 20s with forearm implant Subsequent hypoparathyroidism	US negative CT enlarged left inferior parathyroid	Ptx age 55 3 gland mildly hypercellular 220 810 180 mg IOPTH 55-22 Scan negative US negative CT negative		Cognitive delay
**Patient #**	**11**	**12**	**13**	**14**	**15**
**Age/gender**	29F	56F	54F	35M	64F
**Age initial hypercalcemia noted** **By history or records**	29	13	50	20	59
**FH**	None known	Son	Mother borderline high calcium. Brother with borderline high calcium (has had renal transplant) Sister with pituitary tumor	Adopted	Sister
**Calcium (8.4-10.2 mg/dL)**	10.4-10.7	11.7-13.5^a^	10.1-10.5^b^	11.1-12.7	10.4-11.0
**Albumin (3.8-5.0 g/dL)**	4.5-4.8	4.2-4.7	4.4-4.7^b^	4.4-5.2	4.3-4.6
**Ionized calcium** **(1.18-1.33 mmol/L)**	1.40	1.67, 1.71^a^	1.23-1.37^b^	1.50-1.55	1.29,1.41
**Phosphate (2.5-4.8 mg/dL)**	2.8,2.9	2.0-3.3	2.5,2.9^b^	2.0-2.9	2.7, 2.9
**Magnesium (1.6-2.6 mg/dL)**	2.1	2.3	2.2^b^	2.6-2.8	2.5
**Creatinine (0.5-1.1 mg/dL)**	0.86-0.99	0.86-1.40	0.79-0.93^b^	0.77-1.04	0.72-0.86
**PTH (14-72 pg/dL)**	29, 35	129-158^a^	38-47^b^	29-62	53-61
**25OHD (30-50 ng/dL)**	59, 62	28-64	37-51^b^	10-60	21-38
**Urinary calcium (mg/24 h)**	147	22	126 (after first surgery)	119 (no urinary creatinine), 85 (no urinary creatinine), 292 182 118	129
**CCCR**	0.012	0.002	0.01 (after first surgery)	0.014, 0.013, 0.008	0.008
**BMD** **Lowest T or Z**	Z-score −1.0	T-Score −2.3	T-Score −1.1	Z-Score −2.5	T-Score −1.8
**Kidney stones**	No	No	No	No	No
**Genetic testing**	*CASR* VUS c2656C>GpARG886GLY (other variants at this position pathogenic and clinical FHH patient with same variant in Tennessee (personal communication Bushra Osmani)	Pathogenic variant *AP2S1* c.44G>T (p.Arg15Leu)	VUS *CASR* c.206G>A (p.Arg69His) later upgraded to likely pathogenic	Pathogenic variant *CASR* gene c.680G>A (p.Arg227Gln)	VUS *CASR* c.725T>C (p.Leu242Pro)
**Alpha missense**	Ambiguous				Likely pathogenic
**Other genes tested**	*AP2S1, CDC73, CDKN1B, GCM2, GNA11, MEN1, RET*	*CASR, CDC73, CDKN1B, MEN1, GNA11, RET*	*AP2S1, CDC73, CDKN1B, GNA11, MEN1, RET*	*AP2S1, CDC73, CDKN1B, GCM2, GNA11, MEN1, RET*	
**Treated with cinacalcet**	No	Yes	No	No	No
**Miscellaneous**		Calcium 10.9-12.1 mg/dL On cinacalcet US negative	Age 54 calcium 11.2-11.7 mg/dL, PTH ~150 pg/mL Urinary calcium 334 mg/d 920 mg parathyroid adenoma Mild hypercalcemia with non-suppressed PTH persisted, 2.5 glands removed, no change in calcium Before first surgery, CT and US left inferior adenoma. After first surgery scan negative		
**Patient #**	**16**	**17**	**18**	**19**	**20**
**Age/gender**	55F	78F	68F	40F	68M
**Age initial hypercalcemia noted** **by history or records**	26	71	20s	33	60
**FH**	Multiple family members failed Ptx Hypercalcemia mother, sister, son, daughter, and two maternal aunts, granddaughter Mother of patient 22	None known	Mom, 2 siblings	None known	None known
**Calcium (8.4-10.2 mg/dL)**	12.5^b^	9.9-11.4	9.9-11.0	Before Surgery 10.1-11.0 After Surgery 10.2-10.5	10.1-10.8
**Albumin (3.8-5.0 g/dL)**	4.6^b^	4.2-4.8	3.4-4.4	3.5-4.7	4.2-4.5
**Ionized calcium** **(1.18-1.33 mmol/L)**	1.62^b^	1.35-1.42	1.34,1.43	Before surgery 1.41-1.48 After surgery 1.34	1.31-1.42
**Phosphate (2.5-4.8 mg/dL)**	2.7^b^	2.7-3.4	2.4,2.5	2.2-3.7	2.7
**Magnesium (1.6-2.6 mg/dL)**	2.3^b^	2.3-2.6	1.8	2.3	
**Creatinine (0.5-1.1 mg/dL)**	0.67^b^	0.51-0.89	0.60-1.21	0.75-0.99	1.22
**PTH (14-72 pg/dL)**	98^b^	30-54	36-76	42-90	65-91
**25OHD (30-50 ng/dL)**	44^b^	13-38	25-36	30-57	35, 40
**Urinary calcium (mg/24 h)**	152^b^	78, 48	208	296 (prior to surgery no creatinine done) 156 (after surgery)	135 178
**CCCR**	0.008^b^	0.006, 0.004	0.010	0.012 (after surgery)	0.012
**BMD** **Lowest T or Z**	T-Score −0.7	T-Score −1.7	T-Score −2.2	Z-Score −2.7	
**Kidney stones**	No	No	Yes—uric acid	No	
**Genetic testing**	Pathogenic variant *AP2S1* c.44G>T (p.Arg15Leu)	VUS *CASR* c.2045C>T (p.Pro682Leu)	VUS *GNA11* c.646T>A (p.W216R)	VUS *CASR* c.2369T>G (p.Phe790Cys)	Pathogenic *CASR* C1738_1743del (p.ser580_Ala5-81del)
**Alpha missense**		Likely pathogenic	Likely pathogenic	Likely pathogenic	
**Other genes tested**	*CASR, CDC73, CDKN1B, GCM2, GNA11, MEN1, RET*	*GNA11, AP2S1*	*GNA11, AP2S1*	*AP2S1, CDC73, CDKN1B, GNA11, MEN1, RET*	*GNA11, AP2S1, MEN1, RET, CDC73, CDKN1B*
**Treated with cinacalcet**	Yes Calcium 10.0, 10.1 mg/dL; ionized calcium 1.34, 1.36 mmol/L	No	No	No	No
**Miscellaneous**	Subtotal (3.5 gland) parathyroidectomy w/ left SCM implantation of remaining 0.5 gland age 38 yr	Compound heterozygote with this variant this causes NSHPT	Variant segregates with 2 affected and 1 unaffected siblings ClinVar variant not observed in large populations and in-silico analysis predicts deleterious effect	Right superior Ptx (580 mg) IOPTH 44-36 Persistent hypercalcemia within 2 mo CT/US positive before surgery	
**Patient #**	**21**	**22**	**23**	**24**	**25**
**Age/gender**	26F	41F	51F	76F	24M
**Age initial hypercalcemia noted** **by history or records**	22	28	30	63	18
**FH**	Father, Sister	Daughter, brother, mother, maternal neice and nephew, maternal aunts daughter of patient 16	Unknown	Son, Daughter	“Father’s side”
**Calcium (8.4-10.2 mg/dL)**	11.2-11.9	10.2-12.2	9.9-11.2	10.1-10.7	10.1-11.2
**Albumin (3.8-5.0 g/dL)**	4.7-5.4	3.7-4.3	4.1-4.5	4.0-4.8	4.4-5.3
**Ionized calcium** **(1.18-1.33 mmol/L)**	1.48-1.52	6.54 mg/dL (4.5-5.3)	1.45,1.38	1.40-1.43	1.46
**Phosphate (2.5-4.8 mg/dL)**	2.1-2.5	2.4	3.2	2.3, 3.0	3.4-4.9
**Magnesium (1.6-2.6 mg/dL)**	1.8, 2.2	2.1	2.5	1.9	1.9-2.0
**Creatinine (0.5-1.1 mg/dL)**	0.65-0.98	0.47-0.80	0.81-0.94	0.80-1.40	0.84-1.37
**PTH (14-72 pg/dL)**	29-61	97-143	51,42	24-48	21,34
**25OHD (30-50 ng/dL)**	10-70	10-22	46-85	34-48	32-45
**Urinary calcium (mg/24 h)**	158 129	258	Not done	94	62
**CCCR**	0.011, 0.008	0.010		0.008	0.005
**BMD** **Lowest T or Z**					
**Kidney stones**	No			No	No
**Genetic testing**	VUS *CASR* c.569A>G (pAsp190Gly) Later updated to pathogenic	*AP2S1* pathogenic variant, c.44G>T (p.Arg15Leu).	Pathogenic *CASR* c.73C>T (p.Arg25^*^)	Pathogenic *CASR* c.206G>A (p.Arg69His)	Pathogenic *CASR* c.206G>A (p.Arg69His)
**Alpha missense**					
**Other genes tested**	*AP2S1, CDC73, CDKN1B, CGM2, GNA11, MEN1, RET*	*CASR, CDC73, CDKN1B, CGM2, GNA11, MEN1, RET*	*AP2S1, CDC73, CDKN1B, CGM2, GNA11, MEN1, RET*	*GNA11, AP2S1* *MEN1, RET, CDKN1B* *CDC73, CGM2*	*GNA11, AP2S1* *MEN1, RET, CDKN1B* *CDC73, CGM2*
**Treated with cinacalcet**	No	Brief (side effects)	No	No	No
**Miscellaneous**		Scan negative			Low alkaline phosphatase and pathogenic variant *ALPL* Intracranial germinoma

a Before cinacalcet.

b After second surgery.

Reference ranges are in parentheses.

Scan: parathyroid scan, US: parathyroid US, and CT: parathyroid CT.

Abbreviations: CCCR, calcium to creatinine clearance ratio; IOPTH, intraoperative PTH; NSHPT, neonatal severe hyperparathyroidism; Ptx, parathyroidectomy; SCM, sternocleidomastoid; VUS, variant of unknown significance.

## Results

We report 25 cases of suspected FHH from our endocrine practice (20 FHH1, 1 FHH2, and 4 FHH3). The patients are summarized in Table 1. Case 9 was reported previously because his initial genetic testing was negative; however, subsequent analysis by different methodology found a large pathogenic deletion in *CASR.*^23^ Overall, 6 out of 23 patients (26%) had at least one urinary calcium measurement greater than 200 mg/24 h and 10 others had urinary calcium 100-200 mg/24 h. Only 5 patients had urinary calcium that were always <100 mg/daily. The CCCR was consistently <0.01 in only 10 of 22 patients (45%) and 11 of 24 (46%) had at least one elevated PTH. The family history was positive in 18 of our patients; however, this was sometimes only apparent after requesting the patient obtain calcium levels on family members. After germline testing, 8 of the 25 patients had initial VUS. As above, in the patient with suspected FHH2, the variant segregates with 1 affected and 2 unaffected siblings and Alpha Missense suggests the variant is pathogenic.^22^ Of the 7 VUS *CASR* (FHH1), 2 were later upgraded to pathogenic. Another of the *CASR* VUS causes neonatal severe hyperparathyroidism when a compound heterozygote^24^ suggesting it is pathogenic and Alpha Missense^22^ suggests likely pathogenic. The VUS of patient 11 was seen in another young patient with clinical FHH (personal communication Bushra Osmani). Further, there are other pathogenic variants at this amino acid position.^24^ Alpha Missense considers this an ambiguous variant.^22^ Patient 10 described above is interesting. Her family history is very suggestive of a familial condition and her sister reportedly has a low urinary calcium. In this setting, FHH would seem most likely. Interestingly, Alpha Missense considers the variant likely benign.^22^ More detailed family studies would be useful; however, she has been lost to follow-up. The 2 remaining VUS have clinical presentations consistent with FHH. In one case, a sister has hypercalcemia. In the other case, the hypercalcemia dates back to at least age 33 and hypercalcemia persisted after parathyroid surgery. One of our patients described above had clear evidence of PHPT (patient 13). After an adenoma was excised much milder hypercalcemia persisted and did not improve after a second operation. She subsequently had confirmed FHH1. Patient 19 also had improved but persistent hypercalcemia after an enlarged parathyroid gland was removed and may have had co-existent PHPT and FHH.

Parathyroid imaging was found on 9 patients and is included the table. In patients 13 and 19 who appear to have had co-existent PHPT, imaging was positive before surgery. In patient 13, a parathyroid scan was negative after the first operation. In 2 other patients (5 and 7), CT scanning suggested an enlarged parathyroid gland. Sixteen patients had bone density studies by DXA. Two postmenopausal women had T-scores <−2.5 and 2 younger patients had Z-scores <−2.0.

The gene variant was discovered age 21-78 with a median age of 54. The age first elevated calcium identified (record review or history) was 13-71 with a median of about 34. Two of 4 patients with FHH3 were treated with cinacalcet and in 3 of the 4 FHH3 patients, most calcium levels were >11.5 mg/dL before treatment. One FHH1 patient was treated with cinacalcet. Five out of 25 had unnecessary surgery and 1 patient developed permanent postsurgical hypoparathyroidism.

## Discussion

We report our experience with 25 patients with likely FHH. There are several relevant findings in our patients. The correct diagnosis was often delayed for several years after hypercalcemia was discovered. We confirm that too many patients have unnecessary surgery. The percent of FHH patients having inappropriate surgery is not precisely known; however, in 1980, Marx et al. found 9% of patients referred after unsuccessful parathyroid surgery had FHH.^25^ A study from Denmark in 2007 showed 3/13 (23%) of patients with unsuccessful parathyroid surgery had FHH.^26^ We confirm other studies that show over-reliance 24-h urinary calcium or CCCR results in significant diagnostic error.^2^^,^^7^^,^^10^^,^^27^ We also find that hypercalcemia in some patients is significant enough to benefit from medical therapy with cinacalcet.^28^ As reported by others,^29^ marked hypercalcemia is more common in FHH3. Additionally, we report 1 and possibly a second patient with co-existent PHPT and FHH. We also note that many patients with FHH have intermittent eucalcemia and the finding of prior eucalcemia, especially if the calcium is high normal does not exclude FHH. Another interesting finding is that abnormal parathyroid imaging can occur and should not be used to exclude FHH. Lastly, we report several new variants that are probably pathogenic.

Familial hypocalciuric hypercalcemia is an unusual familial condition which mimics PHPT. The differentiation between these conditions is important because patients with FHH should not be subjected to unnecessary and potentially harmful surgery. FHH is caused by pathogenic variants in *CASR* (FHH1) and 2 downstream proteins in calcium sensing encoded by *GNA11* (FHH2), and *AP2S1* (FHH3).^1^ Bi-allelic pathogenic loss of function variants of *CASR* results in severe neonatal hyperparathyroidism.^1^ Further, gain of function variants of *CASR* and *GNA11* cause autosomal dominant hypocalcemia (ADH type 1 and 2, respectively).^30^ FHH1 appears to account for approximately 65% of FHH.^1^ FHH3 is less common than FHH1 and FHH2 is the rarest.^1^ Some families with apparent FHH appear to have yet undiscovered molecular causes. The recently published *CASR* database and website is a useful tool in managing patients with *CASR* variants.^24^

Familial hypocalciuric hypercalcemia may not be as uncommon as previously thought. In a study of approximately 51 000 people, Dershem et al.^31^ found pathogenic loss-of-function FHH1 variants in 74.1/100 000 and a pathogenic loss-of-function *CASR* variant and hypercalcemia in 40.9/100 000. This is of a similar magnitude to the incidence of PHPT in patients under the age of 50.^32^^,^^33^ Thus in younger patients, FHH may be as common as PHPT.

Traditionally, the diagnosis of FHH was made based on the biochemistry of hypercalcemia typically with a normal (sometimes elevated) PTH, urinary calcium <100 mg/24 h, and CCCR < 0.01. As mentioned earlier, the urinary calcium and CCCR are suboptimal in distinguishing FHH from PHPT.^2^^,^^7^^,^^10^^,^^26^ A recent study that included 44 patients with pathogenic *CASR* variants from the Mayo Clinic, found marked overlap between PHPT and FHH1 in urinary calcium and CCCR.^26^ Our study is very consistent with this finding.

Current guidelines suggest genetic testing for FHH in patients with low urinary calcium/CCCR, family history of hypercalcemia, and young age.^34^ We suggest clinicians review records (more widely available in EMR era) to see if hypercalcemia is acquired. It is also important to consider the fact that a negative initial family history may not be accurate and ask patients to request calcium levels on family members if there is suspicion of a familial condition. We believe genetic testing should also be considered if the clinician cannot prove the hypercalcemia is acquired by record review and in the case of persistent PHPT after surgery by an experienced parathyroid surgeon. All 3 FHH genes should be tested and clinicians should recognize that some families with FHH have yet unknown molecular causes. Clinicians should consider working with a certified genetic counselor who can assist with testing logistics, interpretation of results, familial follow-up, and patient education on familial implications.^35^ Clinicians should also be aware of an FHH mimic, autoimmune hypocalciuric hypercalcemia, a rare condition caused by blocking autoantibodies to the CASR, usually associated with other autoimmune conditions.^36–41^ Some of these patients respond to corticosteroid treatment or calcimimetic medications.^36–41^ FHH may co-exist with PHPT and like our patient, surgical removal of an adenoma may result in reduction in hypercalcemia without normalization.^42–46^ It is not clear whether FHH predisposes to PHPT or this is a chance occurrence. Some of our patients had osteoporosis or bone density lower than expected for age based on densitometric criteria. This could be a chance occurrence or related to the presence of low BMD resulting in evaluation of the PTH-calcium system. We cannot speculate on the relationship between FHH and BMD in these patients.

This study is limited by the fact that we are not able to identify all FHH patients seen in our practice. Further, based on our data, we suspect that some patients with FHH have been labeled as having PHPT.

## Conclusion

Familial hypocalciuric hypercalcemia is more common than many clinicians recognize and the standard biochemical testing is not adequate in distinguishing FHH from PHPT. To avoid unnecessary and potentially harmful surgery, we suggest clinicians have a high index of suspicion and consider broadened use of genetic testing.

## Data Availability

De-identified data is available upon request.
